# Bioequivalence Evaluation of Immediate-Release Empagliflozin/Metformin Fixed-Dose Combination Tablets Under Fed Conditions in Healthy Mexican Subjects and Exploratory Analysis of Metformin Dose Proportionality

**DOI:** 10.3390/ph19081200

**Published:** 2026-07-31

**Authors:** Erika Gabriela Guido Ávila, Alberto Martínez Muñoz, Porfirio de la Cruz Cruz, Omar Emmanuel Hernández Piña, José Trinidad Pérez-Urizar, Abraham Escobedo-Moratilla

**Affiliations:** 1 Asofarma de México S.A. de C.V., Av. Santa Fe 485, Lomas de Santa Fe, Contadero, Cuajimalpa de Morelos, Ciudad de México 05348, Mexico; eguido@adium.com.mx (E.G.G.Á.); ophernandez@adium.com.mx (O.E.H.P.); 2 FS Scientia Pharma S.A. de C.V (Authorized Third Party), Av. Himno Nacional 1330, Jardín, San Luis Potosí 78270, Mexico; alberto.martinez@fsscientiapharma.com (A.M.M.); jpurizar@uaslp.mx (J.T.P.-U.); 3 Doctorado Institucional en Ingeniería y Ciencia de Materiales (DICIM) de la Universidad Autónoma de San Luis Potosí (UASLP), Av. Sierra Leona No. 550, Col. Lomas 2da. Sección, San Luis Potosí 78210, Mexico; a365618@alumnos.uaslp.mx; 4 Facultad de Ciencias Químicas de la Universidad Autónoma de San Luis Potosí, Av. Dr. Nava No. 6, Zona Universitaria, San Luis Potosí 78210, Mexico

**Keywords:** empagliflozin, metformin, type 2 diabetes, bioequivalence, pharmacokinetics

## Abstract

**Background/Objectives:** Fixed-dose combinations (FDCs) improve therapeutic adherence in type 2 diabetes. These studies aimed to evaluate the pharmacokinetics and establish the bioequivalence of four immediate-release empagliflozin/metformin FDC formulations under fed conditions in healthy Mexican subjects. **Methods:** A total of 42 healthy volunteers per study (168 total) were enrolled in four randomized, open-label, two-way crossover, single-dose studies with a 14-day washout period. Formulations were administered orally under standardized fed conditions. Plasma concentrations of both drugs were quantified using a validated liquid chromatography coupled with tandem mass spectrometry (LC-MS/MS) method. **Results:** Across all dose strengths, the 90% confidence intervals (CIs) for the geometric mean ratios of peak plasma concentration (C_max_) and area under the concentration–time curve (AUC_0-t_) for both empagliflozin and metformin were entirely within the 80.00–125.00% bioequivalence acceptance range. The specific 90% CIs for C_max_ and AUC_0-t_ were: Study A (5/850 mg): empagliflozin (90.18–103.16%; 94.37–101.81%), metformin (98.43–107.69%; 97.18–106.62%); Study B (12.5/500 mg): empagliflozin (91.94–102.77%; 93.38–98.51%), metformin (92.58–101.73%; 94.85–103.91%); Study C (12.5/850 mg): empagliflozin (95.47–107.74%; 95.34–101.25%), metformin (94.19–103.59%; 94.06–103.21%); and Study D (12.5/1000 mg): empagliflozin (94.53–107.10%; 98.13–105.66%), metformin (98.89–107.51%; 99.25–109.92%). Regarding safety, no serious adverse events occurred, and all reported events were mild or moderate and resolved completely. **Conclusions:** All evaluated empagliflozin/metformin FDCs successfully demonstrated bioequivalence and a favorable safety profile under fed conditions. These findings fulfill regulatory requirements and support their therapeutic interchangeability in clinical practice. An exploratory power-model analysis indicated a modest trend toward less-than-dose-proportional metformin exposure across the 500–1000 mg range (slope β ≈ 0.77–0.78). NCT07633015, NCT07633054, NCT07633028, and NCT07633041.

## 1. Introduction

Diabetes encompasses a group of metabolic disorders of carbohydrate metabolism characterized by sustained hyperglycemia and its associated complications [1]. This imbalance reflects a failure of normal glucose regulation, characterized by reduced tissue utilization and excessive endogenous glucose production through increased gluconeogenesis and glycogenolysis [2].

The rise in prevalence and mortality from type 2 diabetes (T2D) represents a significant challenge for Mexico, requiring a rapidly increasing need for screening, care, and treatment [3]. This challenge is underscored by alarming statistics: In 2022, T2D was the second leading cause of death in the country, resulting in over 115,000 fatalities. By that year, 18.5% of the Mexican population had T2D (an almost 30% increase since 2006). Moreover, nearly one-third of individuals with T2D were previously undiagnosed. T2D is a multifactorial disease resulting from the interplay between genetic determinants, such as impaired insulin secretion and insulin resistance, and environmental factors, including obesity, physical inactivity, aging, and psychosocial stress [4].

The 2025 American Diabetes Association (ADA) guidelines suggest a multi-targeted pharmacological approach for T2D management, encompassing glucose-lowering, weight loss, and management of kidney and cardiovascular disease [5]. This comprehensive strategy is essential because efficient glycemic control is fundamental to reducing the risk of both micro- and macrovascular complications [6]. Since T2D is a progressive disease, combination therapy is often required to maintain glycemic goals. Traditional approaches recommend the stepwise addition of medications to metformin to sustain target glycated hemoglobin (HbA1c) levels. Furthermore, for individuals with T2D who have established atherosclerotic cardiovascular disease (ASCVD), heart failure, or chronic kidney disease, combination therapy including a sodium–glucose cotransporter-2 (SGLT2) inhibitor has demonstrated significant cardiovascular benefits [6].

To address therapeutic adherence issues associated with polypharmacy, fixed-dose combinations (FDCs) of metformin and SGLT2 inhibitors like empagliflozin have been developed. Empagliflozin is an SGLT2 inhibitor indicated for the treatment of diabetes and is used either as monotherapy or in combination with other therapies [7]. However, formulating these FDCs presents a profound biopharmaceutical challenge. Both empagliflozin [8] and metformin [9] are classified as Biopharmaceutics Classification System (BCS) Class III drugs, characterized by high aqueous solubility and low permeability. For BCS Class III compounds, the rate and extent of absorption are permeability-limited, meaning that any variation in formulation excipients within the FDC could potentially alter passive or transporter-mediated gastrointestinal uptake.

Empagliflozin exhibits rapid absorption, with peak plasma concentrations generally achieved between 1.33 and 3.00 h following oral administration [10]. Following peak levels, plasma concentrations decline biphasically, comprising a rapid distribution phase and a relatively slow terminal elimination phase [11]. It has an apparent volume of distribution (Vd) of 73.84 L at steady state [12]. Metabolism is mediated primarily by glucuronidation rather than cytochrome P450 enzymes. Empagliflozin is a substrate for P-glycoprotein and breast cancer resistance protein (BCRP), exhibiting an elimination half-life (t_1/2_) of approximately 7.47 h [13]. Pharmacodynamically, it inhibits glucose and sodium reabsorption in the proximal renal tubules, inducing glycosuria and natriuresis [14,15].

Metformin has an oral bioavailability of 50% to 60% [16]. Following a single 500 mg dose, peak plasma concentrations are reached at approximately 2.33 h [17]. It is widely distributed with virtually negligible protein binding and a large Vd of 654 L [18]. Metformin does not undergo hepatic biotransformation; it is excreted entirely unchanged via renal tubular secretion, with a t_1/2_ of 11.6 h [19]. It functions by decreasing hepatic glucose production, reducing intestinal glucose absorption, and improving peripheral insulin sensitivity [20,21].

Crucially, concomitant food intake significantly alters the absorption of both active pharmaceutical ingredients. Administration in the fed state reduces empagliflozin’s area under the curve (AUC) by approximately 16% and its C_max_ by roughly 37% [14]. Similarly, food intake decreases metformin’s C_max_ by 40% and AUC by 25%, while prolonging the time to reach peak plasma concentration (t_max_) [22]. Because metformin is associated with gastrointestinal tolerability issues, clinical guidelines mandate that these FDCs be administered with meals. Therefore, conducting bioequivalence trials under fed conditions is the most challenging and clinically relevant setting for evaluating formulation-dependent differences.

Although several bioequivalence studies of empagliflozin/metformin FDCs exist, pharmacokinetic data in Latin American populations, particularly in Mexican subjects, remain limited. Given potential ethnic and population-related pharmacokinetic determinants, region-specific evaluations are scientifically justified to support therapeutic interchangeability. Furthermore, the permeability-limited absorption of both active pharmaceutical ingredients (APIs) and the clinically relevant effect of food on their PK behavior provide additional justification for confirming comparable in vivo performance under fed conditions. Therefore, the present work aimed to characterize the pharmacokinetic profiles and unequivocally establish the bioequivalence of four distinct strengths of empagliflozin/metformin immediate-release FDCs in healthy Mexican volunteers under fed conditions. Additionally, because multiple dose strengths were evaluated, an exploratory assessment of metformin dose proportionality across the 500–1000 mg range was performed using a power model.

## 2. Results

### 2.1. In Vitro Dissolution Profiles

**Figure 1:**
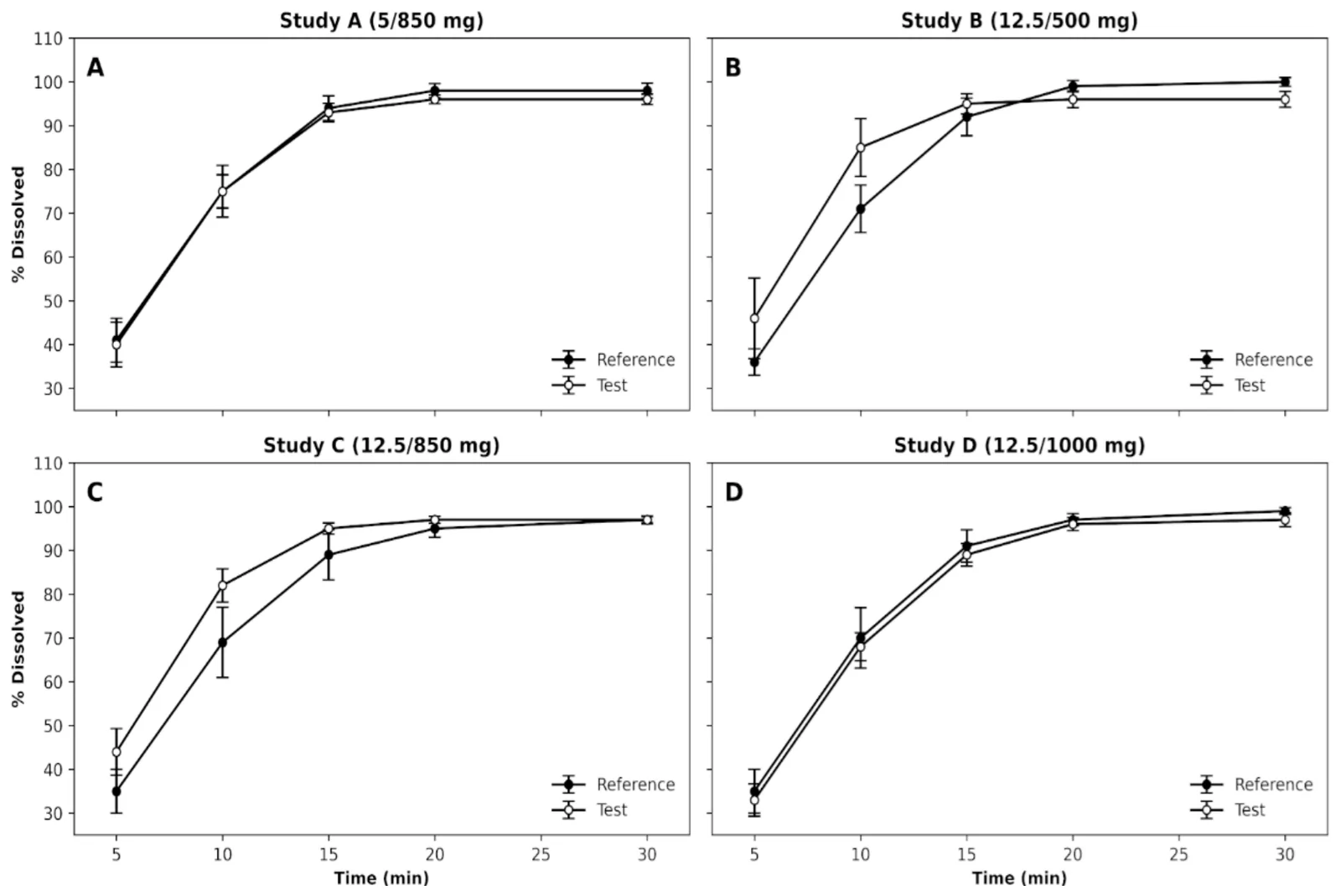
Mean comparative dissolution profiles of empagliflozin from test (white circles) and reference (black circles) fixed-dose combination tablets. The panels represent: (**A**) Study A (5/850 mg), (**B**) Study B (12.5/500 mg), (**C**) Study C (12.5/850 mg), and (**D**) Study D (12.5/1000 mg). Dissolution testing was performed in 900 mL phosphate buffer (pH 6.8) using USP Apparatus II at 50 rpm. Data are presented as mean ± standard deviation (SD) (*n* = 12).

**Figure 2:**
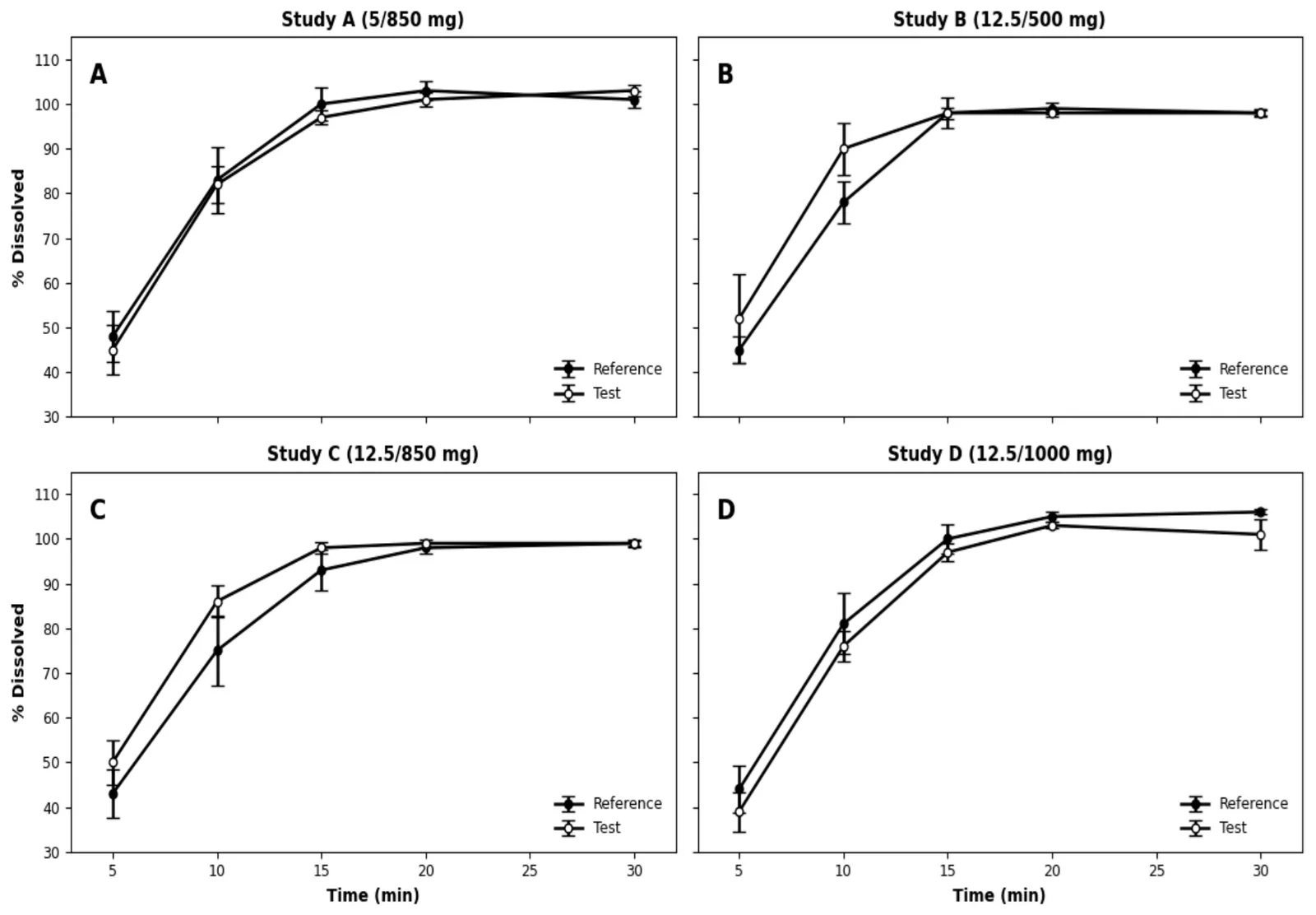
Mean comparative dissolution profiles of metformin from test (white circles) and reference (black circles) fixed-dose combination tablets. The panels represent: (**A**) Study A (5/850 mg), (**B**) Study B (12.5/500 mg), (**C**) Study C (12.5/850 mg), and (**D**) Study D (12.5/1000 mg). Dissolution testing was performed in 900 mL phosphate buffer (pH 6.8) using USP Apparatus II at 50 rpm. Data are presented as mean ± standard deviation (SD) (*n* = 12).

The comparative dissolution profiles of empagliflozin and metformin from the test and reference fixed-dose combination formulations are presented in Figure 1 and Figure 2, respectively. Each figure includes the dissolution profiles corresponding to the four evaluated strengths (5/850 mg, 12.5/500 mg, 12.5/850 mg, and 12.5/1000 mg), which were the same batches assessed in the in vivo bioequivalence studies.

For both empagliflozin and metformin, the test and reference formulations exhibited very rapid dissolution under the experimental conditions. Across all evaluated strengths, at least 85% of the labeled amount was dissolved within 15 min, indicating very rapid and consistent drug release from both products.

According to current regulatory criteria, dissolution profiles that achieve at least 85% dissolution within 15 min are considered similar without requiring a formal comparison using the similarity factor (f2) [23,24]. The very rapid dissolution observed for both APIs indicates that drug release from the dosage form is unlikely to represent a limiting factor for product performance. The close agreement between the test and reference dissolution profiles was consistent with the in vivo bioequivalence demonstrated across all four studies.

For metformin specifically, previous mechanistic absorption modeling studies have demonstrated that C_max_ and AUC are not significantly affected when complete drug release occurs within 2 h after administration, a finding subsequently confirmed with clinical pharmacokinetic data [25]. These investigations further suggest that metformin absorption is primarily governed by permeability rather than dissolution rate [25]. Moreover, in vitro and in silico studies have shown that metformin formulations with different dissolution rates may still yield comparable pharmacokinetic profiles and bioequivalence outcomes, provided rapid, complete drug release is achieved [26]. In the present study, all metformin-containing drug products achieved at least 85% dissolution within 15 min, representing a substantially faster release rate than the conditions previously associated with preserved bioequivalence [25].

Notably, the compendial dissolution conditions employed in this study (USP Apparatus II, phosphate buffer pH 6.8, 50 rpm) produced highly comparable dissolution profiles for the test and reference products across all evaluated strengths. Similar experimental conditions have been reported to provide bio-predictive information regarding the in vivo performance of metformin-containing formulations [26]. Furthermore, successful in vitro–in vivo correlation (IVIVC) models have been developed for modified-release metformin formulations using dissolution testing performed under USP Apparatus II conditions in phosphate buffer pH 6.8 [27]. It should be noted that the development or evaluation of an IVIVC was beyond the scope of the present study. Importantly, the IVIVC models reported in the literature were developed for modified-release metformin formulations and therefore cannot be directly extrapolated to the immediate-release products evaluated herein. Nevertheless, the consistency between the dissolution findings and the observed bioequivalence outcomes supports the relevance of the dissolution methodology used. Overall, these results provide additional pharmaceutical evidence supporting the in vivo bioequivalence demonstrated across all four evaluated strengths.

### 2.2. Demographic Characteristics

A total of 42 healthy Mexican volunteers were enrolled in each of the four independent bioequivalence studies. The baseline demographic characteristics of the participants, including gender distribution, age, weight, height, and body mass index (BMI), are comprehensively summarized in Table 1.

Overall, the demographic profiles were consistent across the four cohorts. Mean ages ranged from 35.00 ± 11.00 years (Study B) to 37.90 ± 12.30 years (Study D). Similarly, mean BMI was highly comparable among the studies, ranging from 23.30 ± 2.72 kg/m^2^ (Study C) to 24.30 ± 2.00 kg/m^2^ (Study A). Overall, the consistency observed in baseline demographic characteristics supported the comparability of the study population before dosing.

**Figure 3:**
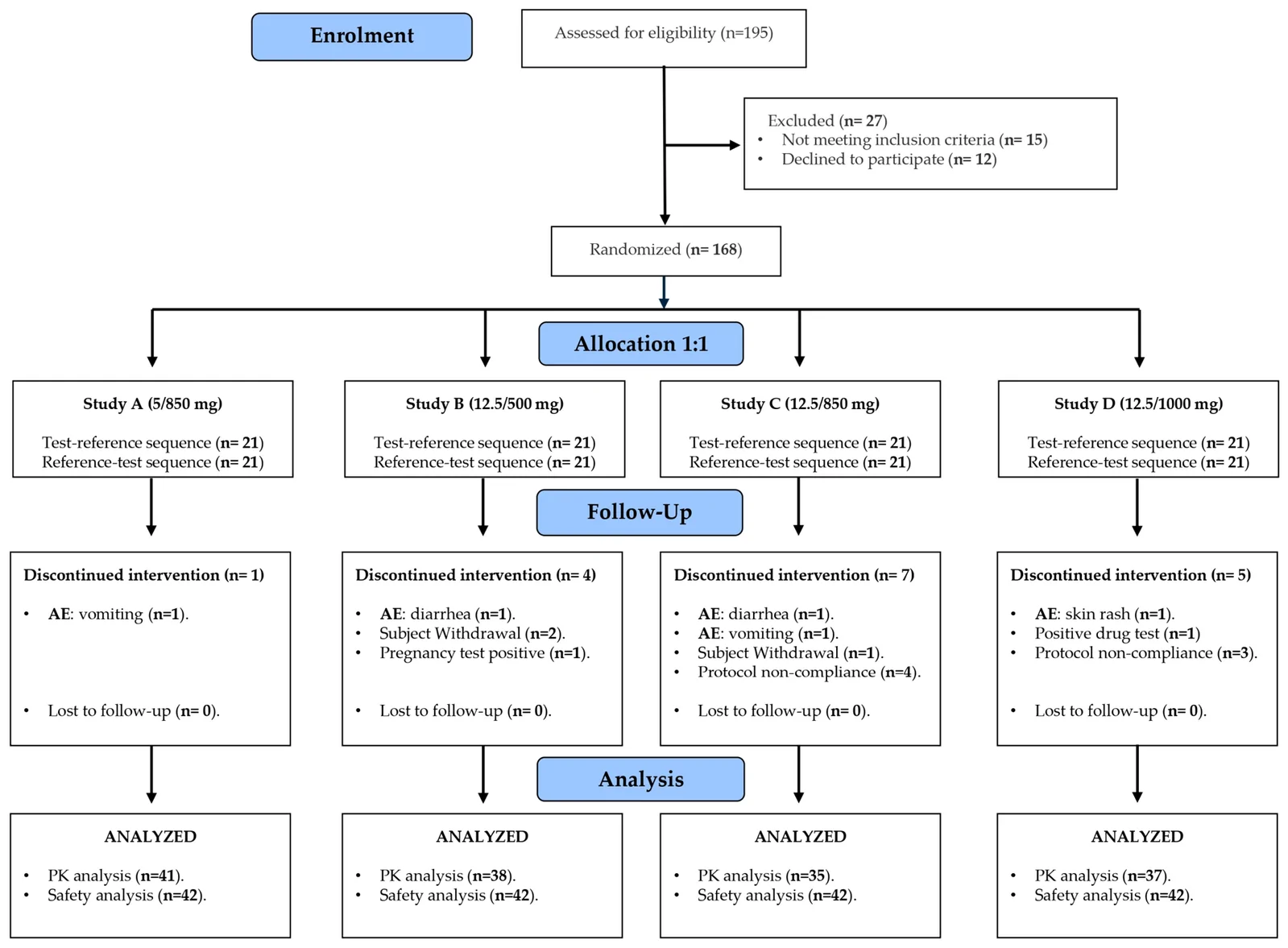
Consolidated CONSORT flow diagram depicting subject disposition (screening, randomization, follow-up, and analysis) across the four independent clinical studies. AE: adverse event; n: number of subjects; PK: pharmacokinetic.

Regarding study completion, while 42 subjects initiated each trial, some participants withdrew during the clinical phase. A detailed breakdown of subject screening, randomization, sequence allocation, and follow-up for all four cohorts is presented in the consolidated CONSORT flow diagram (Figure 3). The studies were successfully finalized by 41, 38, 35, and 37 volunteers for Studies A, B, C, and D, respectively. Subjects who completed the study constituted the per-protocol (PP) population utilized for all subsequent pharmacokinetic (PK) and statistical analyses.

### 2.3. Pharmacokinetics

**Figure 4:**
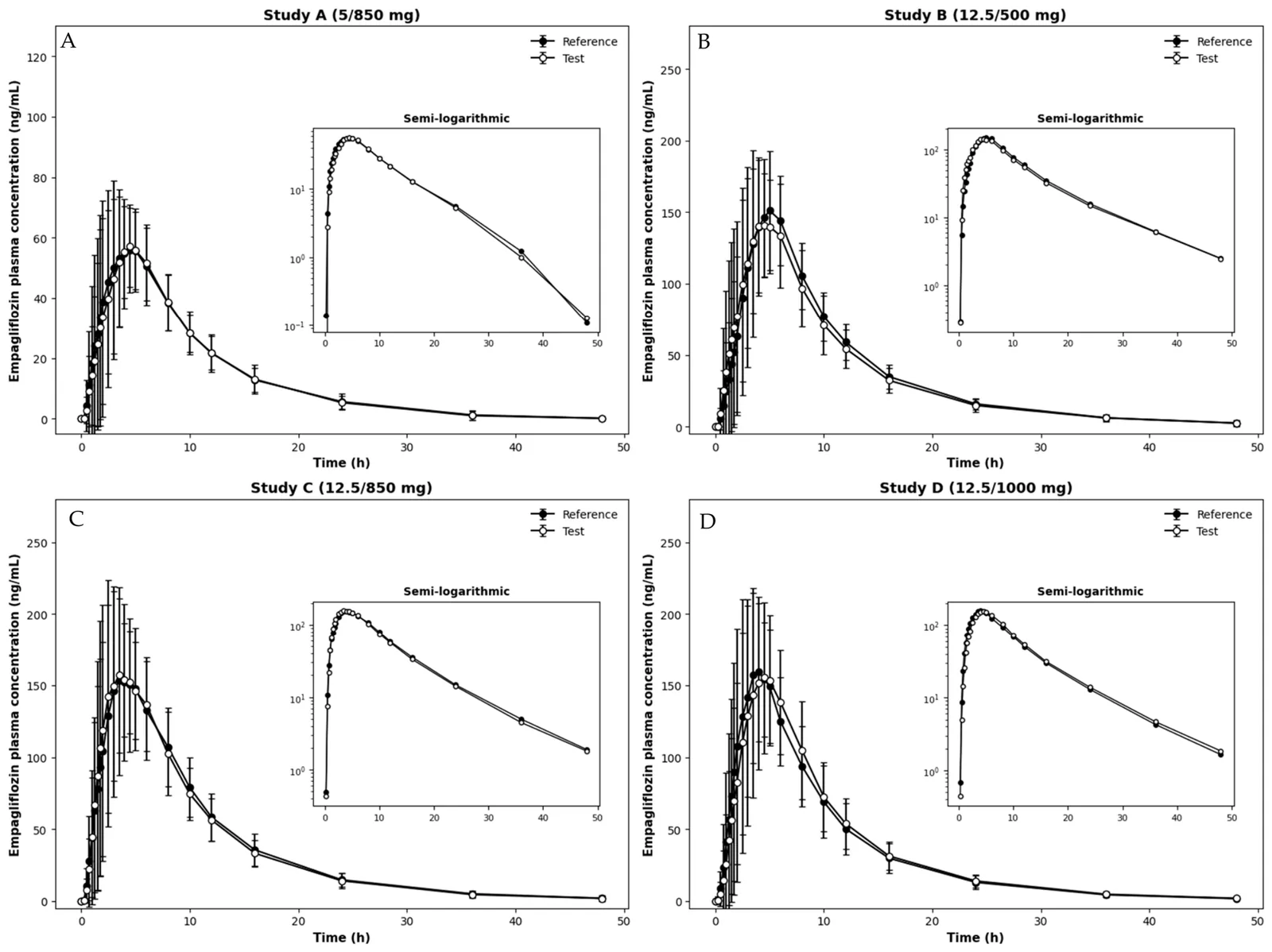
Mean plasma concentration–time profiles of empagliflozin following a single oral administration of test (white circles) and reference (black circles) fixed-dose combination tablets under fed conditions. The panels represent: (**A**) Study A (5/850 mg), (**B**) Study B (12.5/500 mg), (**C**) Study C (12.5/850 mg), and (**D**) Study D (12.5/1000 mg). Data are presented as mean ± standard deviation (SD). The inset in each panel depicts the same pharmacokinetic profiles on a semi-logarithmic scale to illustrate the terminal elimination phase.

**Figure 5:**
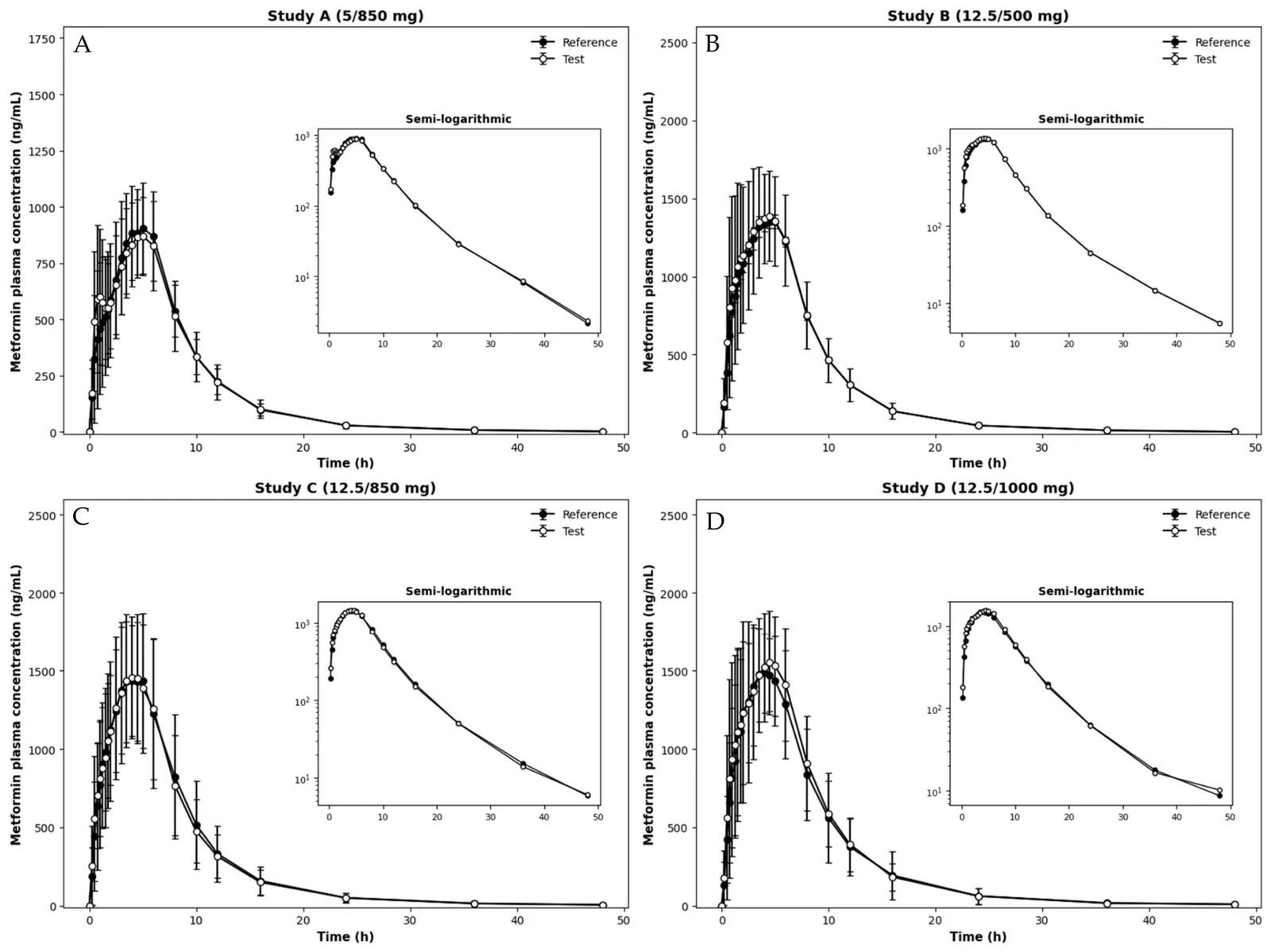
Mean plasma concentration–time profiles of metformin following a single oral administration of test (white circles) and reference (black circles) fixed-dose combination tablets under fed conditions. The panels represent: (**A**) Study A (5/850 mg), (**B**) Study B (12.5/500 mg), (**C**) Study C (12.5/850 mg), and (**D**) Study D (12.5/1000 mg). Data are presented as mean ± standard deviation (SD). The inset in each panel depicts the same pharmacokinetic profiles on a semi-logarithmic scale to illustrate the terminal elimination phase.

The mean plasma concentration–time profiles (Cp vs. t) for both empagliflozin and metformin following administration of the test and reference fixed-dose combinations (FDCs) were virtually superimposable across all four studies under fed conditions (Figure 4 and Figure 5).

The detailed primary and secondary pharmacokinetic parameters C_max_, t_max_, AUC_0-t_, AUC_0-∞_, and t_1/2_ for both APIs are comprehensively summarized in Table 2. Across all four evaluated dosage strengths (Studies A–D), the peak systemic exposures and total extent of absorption were similar between the test generic formulations and their respective reference drug product.

To formally establish bioequivalence, 90% confidence intervals (CIs) for the geometric mean ratios (test/reference) were calculated for the log-transformed primary parameters, C_max_ and AUC_0-t_. As presented in Table 3, all evaluated metrics met the regulatory requirements. For empagliflozin, the 90% CIs across all four studies ranged from 90.18% to 107.74% for C_max_ and from 93.38% to 105.66% for AUC_0-t_.

For metformin, the 90% CIs across all four studies ranged from 92.58% to 107.69% for C_max_, and from 94.06% to 109.92% for AUC_0-t_.

Because every calculated 90% CI for both compounds and all formulations fell within the predefined regulatory acceptance margins of 80.00% to 125.00%, the bioequivalence of the four generic empagliflozin/metformin FDCs to the reference products was clearly demonstrated. Statistical analysis was performed using analysis of variance (ANOVA) on the log-transformed primary pharmacokinetic parameters to evaluate the effects of sequence, period, and formulation.

No statistically significant effects were observed for sequence, period, or formulation factors (*p* > 0.05), confirming the validity of the study design and the robustness of the bioequivalence assessment.

The rapid in vitro dissolution of both active ingredients (exceeding 85% within 15 min) provides additional pharmaceutical support for the consistent in vivo performance observed. The low intra-subject variability across the clinical phases—for instance, maximum CV_intra_ ranging from 10.60% to 13.00% for metformin AUC_0-t_, and 6.90% to 18.20% for empagliflozin—contributed to the precise estimation of pharmacokinetic parameters and the narrow 90% confidence intervals observed across all four dosage strengths. Taken together, these findings are consistent with reproducible systemic exposure and support the superimposable plasma concentration–time curves observed for the test and reference formulations.

An exploratory evaluation of metformin dose proportionality across the evaluated dose range (500–1000 mg) was conducted on the reference formulations. Mean C_max_ and AUC_0-t_ increased with dose, but the increase was less than dose-proportional, as evidenced by decreasing dose-normalized exposure: dose-normalized C_max_ and AUC_0-t_ differed by approximately 17.4% and 13.8%, respectively, between the lowest and highest doses (Table 4).

**Figure 6:**
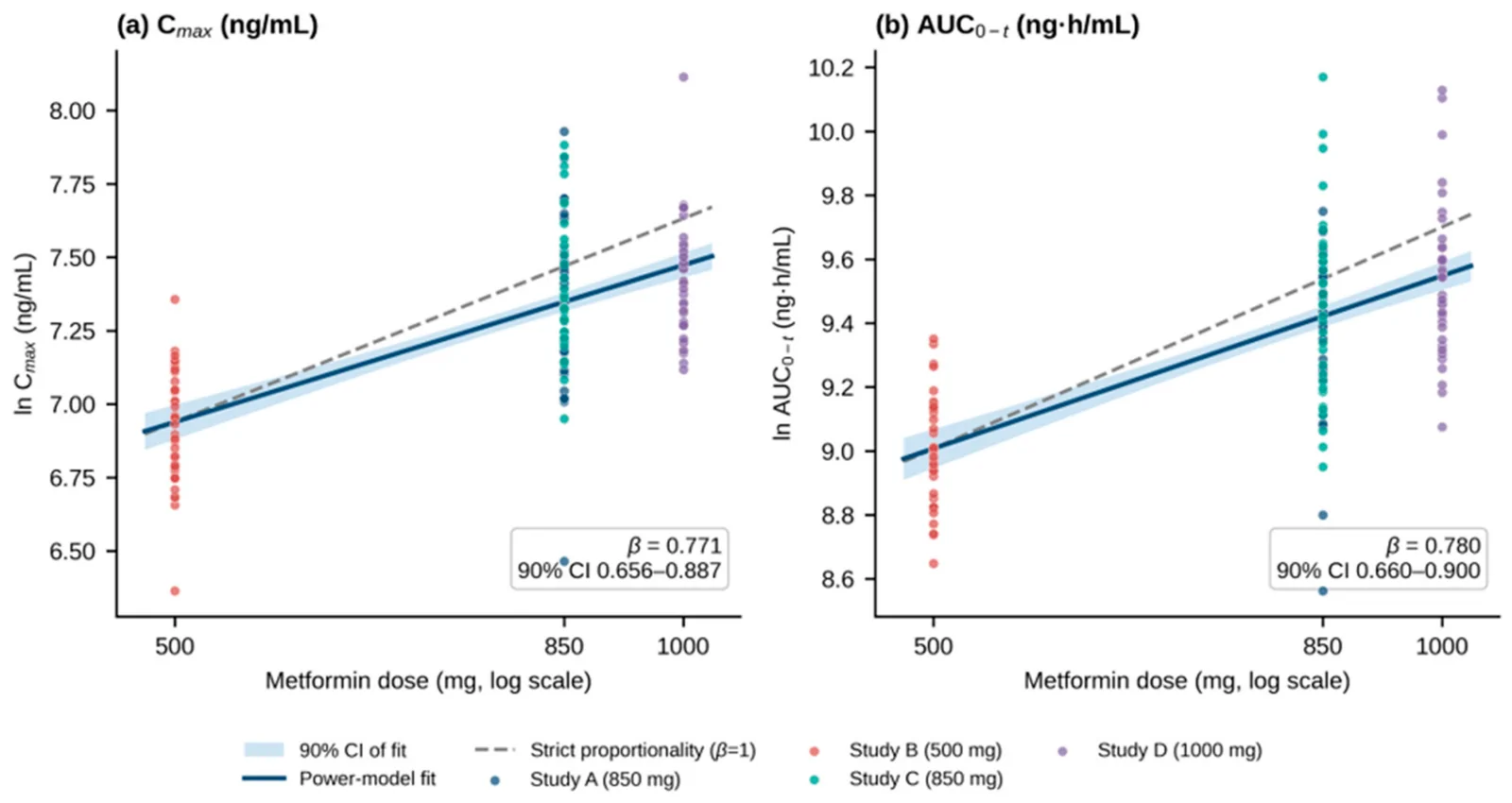
Power-model assessment of metformin dose proportionality across the 500–1000 mg range, based on the reference formulations. (**a**) ln C_max_ and (**b**) ln AUC_0-t_ versus ln dose, with the ordinary least-squares power-model fit (solid line), its 90% confidence interval (shaded), and the line of strict dose proportionality (β = 1; dashed). Individual reference observations are shown by study (Study B, 500 mg; Studies A and C, 850 mg co-administered with 5 and 12.5 mg empagliflozin, respectively; Study D, 1000 mg). Slope estimates β with 90% CIs are inset.

The power model confirmed a less-than-dose-proportional relationship, with slope estimates of β = 0.771 (90% CI 0.656–0.887) for C_max_, β = 0.780 (90% CI 0.660–0.900) for AUC_0-t_, and β = 0.768 (90% CI 0.648–0.888) for AUC_0-∞_ (Figure 6, Table 5). In each case, the 90% CI of β lay below unity and was not entirely contained within the proportionality acceptance region [0.678, 1.322] derived for the 2-fold dose range, indicating that strict dose proportionality was not met. A sensitivity analysis excluding Study C (12.5/850 mg) yielded consistent estimates (β = 0.748, 0.766, and 0.754 for C_max_, AUC_0-t_, and AUC_0-∞_, respectively).

### 2.4. Safety and Tolerability Analysis

In all four clinical studies (A–D), the test and reference fixed-dose combination tablets were generally well-tolerated. A total of 94 adverse events (AEs) were reported throughout the clinical program, of which 49 occurred in the test group and 45 in the reference group. Crucially, no serious adverse events (SAEs) occurred. All AEs were classified as either mild (severity grade 1) or moderate (severity grade 2) in intensity, with complete resolution, no sequelae or lasting damage, and the majority were categorized as probable or possible in relation to the study medications.

As summarized in Table 6, the frequency and distribution of AEs were comparable between the test and reference treatment arms in most studies, except in Study D, where the test group had a higher AE frequency. Although a higher incidence of adverse events was observed in the test arm of Study D, no consistent pattern or clinically relevant differences were identified, and events were consistent with the known safety profile of the individual components.

In terms of demographic distribution, AEs were reported more frequently among female subjects than among male subjects throughout the clinical program. The most common adverse events observed were headache, nausea, dyspepsia, diarrhea, and vomiting. The distribution and AE profiles were consistent with the known safety profile of empagliflozin and metformin, with no clinically meaningful differences between the test and reference formulations. A limited number of subjects required pharmacological intervention (such as ondansetron, ketorolac, or loperamide) to resolve their symptoms.

Participant withdrawals and eliminations during the clinical phase were rigorously documented to ensure the integrity of the per-protocol population. The specific reasons for subject discontinuation across the studies were as follows: Study A: One subject was withdrawn due to an AE, vomiting. Study B: Four subjects were eliminated: one due to an AE (diarrhea), two voluntarily withdrew their informed consent, and one was eliminated after meeting an exclusion criterion (positive pregnancy test before dosing). Study C: Seven subjects were discontinued: one voluntarily withdrew informed consent, four were eliminated for protocol non-compliance (failure to consume the standardized high-fat breakfast), and two were excluded following vomiting or diarrhea. Study D: Five subjects were discontinued for safety or protocol deviations: one due to an AE (skin rash), three due to failure to consume the standardized high-fat breakfast, and one because of a positive drug screening test. All subjects withdrawn due to adverse events were closely monitored by clinical staff until their symptoms were resolved completely. These participants were excluded from the final pharmacokinetic and statistical analyses in accordance with the study protocol and applicable regulatory guidelines.

## 3. Discussion

Metformin is the widely accepted first-line drug for T2DM and the only available biguanide [28]. It is effective both as monotherapy and in combination, with proven beneficial effects on microvascular and macrovascular complications of DM [29]. When patients are not adequately controlled with monotherapy (either empagliflozin or metformin), the fixed-dose combination (FDC) of empagliflozin/metformin hydrochloride can be used alongside diet and exercise. Clinical data indicate that this combination can improve glycemic control in T2DM and reduce body weight and blood pressure compared with each agent alone, within a manageable risk profile [28]. It is especially beneficial for patients inadequately controlled on metformin who have risk factors for cardiovascular disease, declining renal function, or who would benefit from modest reductions in blood pressure and body weight [28,29]. The availability of empagliflozin/metformin as a single-pill combination offers important therapeutic and practical advantages for the treatment of T2D. Because multiple metabolic abnormalities characterize T2DM, many patients require combination therapy with glucose-lowering agents acting through complementary mechanisms to achieve and maintain glycemic targets. Empagliflozin and metformin provide complementary mechanisms of action and have demonstrated efficacy, safety, and tolerability when used in combination [28,29]. Furthermore, the single-pill formulation reduces pill burden and simplifies dosing regimens compared with taking individual tablets, which may improve treatment adherence [28,29]. Given the important therapeutic and practical advantages of the empagliflozin/metformin fixed-dose combination for the treatment of T2D, the bioequivalence demonstrated in this work supports the therapeutic equivalence and interchangeability of the evaluated generic formulations. Across the four dosage strengths evaluated under fed conditions in healthy Mexican subjects, the primary pharmacokinetic parameters, C_max_ and AUC_0-t_, for both analytes met the regulatory acceptance criteria (Studies A–D).

The consistency of these pharmacokinetic findings can be interpreted through the lens of the Biopharmaceutics Classification System (BCS). Both empagliflozin and metformin are classified as BCS Class III compounds, characterized by high solubility and low intestinal permeability [30,31]. Because the absorption of BCS Class III drugs is primarily limited by permeability rather than dissolution, these compounds are generally considered more susceptible to formulation-related factors, including excipients that may influence intestinal transport processes [32].

This consideration is particularly relevant for the present FDC, as metformin absorption is mediated by organic cation transporters (OCT1, OCT2) and PMAT [33], while empagliflozin is a substrate for P-glycoprotein (P-gp) [13]. Moreover, transporter-mediated efflux has been recognized as a potential determinant of oral absorption for low-permeability drugs [34]. Despite these theoretical concerns, highly comparable pharmacokinetic profiles were observed across the four evaluated fixed-dose combination strengths (5/850 mg, 12.5/500 mg, 12.5/850 mg, and 12.5/1000 mg). In addition, both the test and reference formulations exhibited very rapid dissolution (pH 6.8), achieving at least 85% drug release within 15 min for empagliflozin and metformin across all evaluated strengths.

This finding is especially important for metformin, for which mechanistic absorption modeling and clinical pharmacokinetic studies have demonstrated that systemic exposure remains largely unaffected when complete drug release occurs within considerably longer (2 h) time frames than those observed in the present research. These findings suggest that metformin absorption is primarily governed by permeability rather than dissolution rate. Therefore, the very rapid and highly comparable dissolution profiles observed for both active ingredients provide additional pharmaceutical support for the demonstrated in vivo bioequivalence.

The robust bioequivalence demonstrated in our cohorts aligns closely with recent literature evaluating this fixed-dose combination. The intra-subject variabilities observed in our study (6.90–18.20% for empagliflozin and 10.60–13.00% for metformin) are highly consistent with the fed-state bioequivalence study conducted by Reza et al. ([35], which reported comparable CVintra values for both empagliflozin (7.85–16.10%) and metformin (13.52–21.26%).

Both empagliflozin and metformin are classified as Biopharmaceutics Classification System (BCS) Class III compounds, meaning their rate and extent of absorption are primarily permeability-limited rather than dissolution-limited [30,31]. Our findings demonstrate that the specific excipients utilized in these fixed-dose combinations (FDCs) do not interfere with the gastrointestinal transport mechanisms of either active pharmaceutical ingredient [32]. Furthermore, a direct comparison of the key pharmacokinetic parameters from our 12.5/1000 mg cohort (Study D) with existing studies further supports the consistency of the PK performance and validates the reliability of these formulations. Under fasting conditions, Pena et al. [36] reported a relatively short median t_max_ of approximately 2.0 h for empagliflozin and 2.5 h for metformin. Consistent with the well-documented food effect, our fed-state study demonstrated a predictable, prolonged t_max_ of approximately 4.10 h for empagliflozin and 4.04 h for metformin. This delayed absorption closely matches the fed-state findings by Reza et al. [35], where t_max_ was similarly delayed to 3.18–3.67 h for empagliflozin and 2.98–3.45 h for metformin, without compromising the total extent of exposure. Regarding peak concentration and total extent of absorption, our findings for the 12.5/1000 mg dose (empagliflozin C_max_~194.78 ng/mL, AUC_0-t_~1651.53 ng·h/mL; metformin C_max_~1768.30 ng/mL, AUC_0-t_~14,834.58 ng·h/mL) are highly comparable in magnitude to those reported by Reza et al. [35] (metformin C_max_~1610 ng/mL, AUC_0-t_~12,151 ng·h/mL) and Pena et al. [36] (metformin C_max_ ~1617 ng/mL, AUC_0-t_~12,563 ng·h/mL), confirming the robustness of the evaluated formulations.

In addition to the bioequivalence assessment, an exploratory power-model analysis of metformin across the 500–1000 mg dose range revealed a modestly less-than-dose-proportional increase in systemic exposure, with power-model slopes consistently below unity (β≈0.77−0.78) and 90% CIs falling partly below the proportionality acceptance region [0.678, 1.322]. While mean C_max_ and AUC_0-t_ increased with dose, dose-normalized exposures decreased at higher doses. This observation provides critical in vivo mechanistic insight into the saturable intestinal uptake of metformin, a well-documented phenomenon [37,38,39]. Metformin absorption relies heavily on transporter-mediated pathways in the enterocyte, predominantly the organic cation transporters (OCT1, OCT2) and the plasma membrane monoamine transporter (PMAT) [33,40,41]. As the administered oral dose increases toward 1000 mg, these transporter systems approach saturation, leading to a decreased fractional absorption [40]. Clinically, this intrinsic pharmacokinetic characteristic indicates that doubling the dose will yield a slightly lower-than-doubled systemic exposure. Importantly, this saturable absorption is inherent to the active molecule itself and does not reflect a transporter limitation or a defect in the formulation, ensuring that generic products perform exactly as expected physiologically across the therapeutic dose range.

For reference, ICH M13A considers pharmacokinetics dose-proportional when the difference in dose-adjusted mean C_max_ and AUC is no more than 25%; in the present data, the dose-normalized differences (≈17.4% for C_max_ and ≈13.8% for AUC_0-t_) remained below this regulatory threshold, even though the more stringent power-model criterion was not met [42]. Importantly, this exploratory observation does not affect the bioequivalence conclusions, as each strength was independently evaluated against its corresponding reference, and the slight non-proportionality was observed in both the test and reference products.

Because the present studies were not specifically designed to evaluate dose proportionality, these findings should be interpreted as descriptive and exploratory rather than as a formal characterization of metformin pharmacokinetics. Importantly, this observation does not compromise the bioequivalence conclusions, as each strength was independently evaluated against its corresponding reference formulation.

Taken together, these results suggest that neither the formulation composition nor the combined administration of both active ingredients produced clinically meaningful alterations in transporter-mediated absorption or systemic exposure, supporting the robustness of the fixed-dose combination strategy. The consistent pharmacokinetic performance observed across the four evaluated strengths indicates no formulation-dependent differences in systemic exposure. Although the exploratory comparison of dose-normalized exposure suggested a less-than-dose-proportional increase in metformin exposure across dose levels, this behavior is a known characteristic of metformin and was observed equally within the context of bioequivalent formulations.

Regarding metabolic disposition, empagliflozin is primarily eliminated via uridine diphosphate glucuronosyltransferase (UGT)-mediated glucuronidation, with minimal involvement of the cytochrome P450 (CYP450) system. At the same time, metformin undergoes no hepatic metabolism and is excreted unchanged. The uniformity in our pharmacokinetic parameters suggests that interindividual variability in drug disposition was effectively controlled by the crossover design. These results are consistent with previously reported data [35,36], supporting the robustness of the present findings and suggesting minimal ethnic variability in the disposition of these compounds when administered as FDCs.

Bioequivalence was formally established as all 90% confidence intervals (CIs) for the geometric mean ratios (test/reference) fell within the accepted 80–125% range, fulfilling COFEPRIS regulatory requirements. Furthermore, ANOVA confirmed that no significant effects of formulation, period, or sequence were detected for C_max_, AUC_0-t_, and AUC_0-∞_ (*p* > 0.05).

Regarding safety, the profile observed in our subjects—characterized primarily by mild to moderate gastrointestinal and neurological events such as headache, nausea, dyspepsia, and loose stools—is consistent with previously published data. While Pena [36] reported an absence of adverse events under fasting conditions, Reza [35] documented mild gastrointestinal events (such as diarrhea and vomiting) when the combination was administered under fed conditions, which mirrors the tolerability observations in our present cohorts. Maintaining this predictable and favorable tolerability profile is of critical clinical relevance for ensuring long-term patient adherence. It provides additional evidence of the safety of these fixed-dose combinations in a healthy Mexican population.

The validation of therapeutic interchangeability for these formulations carries significant clinical and public health implications, particularly for Latin American populations [3]. The management of type 2 diabetes often requires aggressive, multi-targeted pharmacological approaches [5]. By replacing loose-pill regimens with a single fixed-dose combination, the daily pill burden is substantially reduced, a primary driver of improved long-term patient adherence and treatment compliance [28,29]. Furthermore, establishing unequivocal bioequivalence under the stringent regulatory requirements of COFEPRIS [24] facilitates confident generic substitution. This regulatory validation ensures that healthcare systems in Mexico and the broader Latin American region can provide expanded access to cost-effective, high-quality, and reliable therapies, ultimately optimizing glycemic control and reducing the long-term economic and clinical burden of diabetic complications [3,28].

This study has some limitations. Like most bioequivalence studies, it was conducted in healthy volunteers under single-dose conditions, which may not fully reflect the pharmacokinetic behavior, steady-state accumulation, or potential alterations in clearance present in patients with type 2 diabetes receiving chronic therapy. Nevertheless, demonstrating strict bioequivalence ensures that the generic formulations deliver equivalent systemic exposure to the reference products. Consequently, despite the healthy-volunteer setting, clinicians can confidently expect comparable therapeutic efficacy and safety profiles when prescribing these fixed-dose combinations for the long-term management of type 2 diabetes. Additionally, the evaluation was performed exclusively under fed conditions; therefore, extrapolation to fasting conditions should be made with caution. Furthermore, although both male and female participants were included across all studies, the sample sizes were strictly powered to demonstrate overall bioequivalence and were not explicitly designed to detect potential sex-based pharmacokinetic differences, thereby precluding formal exploratory analyses by gender.

Finally, although a power-model analysis of metformin dose proportionality was performed, these observations must be interpreted with caution. The analysis relied on pooling data from parallel cohorts of different subjects rather than utilizing a formal dose-ranging crossover design within a single cohort. Furthermore, the co-administration of different empagliflozin strengths (5 mg vs. 12.5 mg) across these cohorts introduces a methodological consideration, even though no clinically relevant pharmacokinetic interaction is expected between these two agents [43]. Additionally, the narrow 2-fold dose range (500–1000 mg) further limits the precision of the slope estimate and widens the proportionality acceptance region, underscoring that these findings are exploratory.

Overall, the present work provides robust pharmacokinetic and regulatory evidence supporting the bioequivalence, safety, and interchangeability of four empagliflozin/metformin fixed-dose combination formulations. The exploratory analysis of dose-normalized metformin exposure provides additional context regarding exposure trends across clinically relevant dose levels, while the primary findings consistently demonstrate bioequivalence across all evaluated strengths. These findings are particularly relevant for regulatory decision-making in Latin American populations, where locally generated pharmacokinetic data remain limited, ultimately supporting broader access to cost-effective and reliable generic therapies.

## 4. Materials and Methods

### 4.1. Drug Products

The test drug products consisted of immediate-release (IR) empagliflozin/metformin fixed-dose combination (FDC) tablets in four dosage strengths (5 mg/850 mg, 12.5 mg/500 mg, 12.5 mg/850 mg, and 12.5 mg/1000 mg), manufactured by Asofarma de México S.A. de C.V. (Mexico City, Mexico). The reference formulation was manufactured by Boehringer Ingelheim (Burlington, ON, Canada) Ltd. and was obtained from the authorized market.

Batch numbers, manufacturing dates, and expiry dates were verified for all products before administration. The review of the Certificate of Analysis (CoA) confirmed that each test batch complied with the manufacturer’s specifications, as required by NOM-177-SSA1-2013 for products used in bioequivalence studies [24]. All investigational products were stored under controlled conditions, according to the manufacturer’s labeled instructions, until administration to study subjects.

### 4.2. In Vitro Dissolution Studies

Comparative in vitro dissolution profiles were obtained for the test and reference empagliflozin/metformin fixed-dose combination tablets corresponding to the same batches evaluated in the in vivo bioequivalence studies. The evaluated strengths were 5/850 mg, 12.5/500 mg, 12.5/850 mg, and 12.5/1000 mg.

The dissolution experiments were carried out using USP Apparatus II (Elite 8 Dissolution Tester, Hanson Research, Chatsworth, CA, USA) at 50 rpm with 900 mL of phosphate buffer (pH 6.8) kept at 37.0 ± 0.5 °C. Twelve tablets of each test and reference formulation were evaluated. Samples were collected at predefined time points without medium replacement and filtered through 0.45 µm PES Titan3 membrane filters before analysis.

HPLC-UV quantified the amount of dissolved drug at 240 nm. The dissolution conditions were selected in accordance with recommendations described in the FDA Dissolution Methods Database [44], the Mexican Pharmacopeia (FEUM 13.0) [45], and the criteria established in NOM-177-SSA1-2013 [24] for comparative dissolution profile studies used in interchangeability assessments.

The mean percentage dissolved and the standard deviation were calculated for empagliflozin and metformin at each sampling time.

### 4.3. Study Design

The study protocol and informed consent form were reviewed and approved by an independent Research and Ethics Committee and the Mexican Ministry of Health’s Federal Commission for the Protection against Sanitary Risks (COFEPRIS). The research was performed in strict accordance with the Declaration of Helsinki, amended by the 75th WMA General Assembly (Helsinki, Finland, October 2024) and the International Council for Harmonization (ICH) Good Clinical Practice (GCP) guidelines. All participants provided written informed consent before undergoing any study-related procedures, and the trial was managed in full compliance with applicable local regulatory requirements.

Because the formulation components do not maintain strict dose proportionality across the four evaluated strengths (5/850 mg, 12.5/500 mg, 12.5/850 mg, and 12.5/1000 mg), current regulatory frameworks mandate independent bioequivalence evaluations for each specific fixed-dose combination strength against its respective reference product [24].

This single-center, single-dose, open-label, randomized, two-way crossover, prospective, and longitudinal study was conducted under fed conditions to account for the known food effect on metformin pharmacokinetics. To ensure standardization, all subjects underwent at least 10 h of overnight pre-dose fasting on the study day. Participants then received a standardized high-fat, high-calorie breakfast (approximately 800 kcal). Dosing was administered 30 min after the start of this meal, followed by the intake of a 10% glucose solution (250 mL). The glucose solution was administered as a prophylactic measure to reduce the risk of hypoglycemia. It was not intended to simulate fed-state caloric intake beyond the standardized meal, in accordance with institutional clinical practice.

The administration of the fixed-dose combinations under fed conditions was chosen both to comply with established regulatory guidance for metformin-containing products, which recommends administration with meals to improve gastrointestinal tolerability (such as FDA recommendations [46]) and to account for the pharmacokinetic characteristics of the combination [22]. Food intake significantly alters the absorption profile of metformin and empagliflozin, decreasing peak concentrations and delaying absorption. At the same time, clinical guidelines strictly mandate administering these agents with meals to mitigate common gastrointestinal tolerability issues [14,22]. Therefore, evaluating bioequivalence in the fed state represents the most clinically relevant and challenging condition for assessing formulation performance [22].

Treatments were assigned following a computer-generated randomization schedule to ensure sequence balance [24]. A 14-day washout period was implemented between treatment periods [24]. This duration is fully justified, as it safely exceeds seven times the terminal elimination half-lives of both active ingredients—approximately 7.47 h for empagliflozin [13] and 11.6 h for metformin [19]—thereby ensuring complete systemic clearance and preventing potential carryover effects between study periods [24].

Axis Clinical Latina, S.A. de C.V. (Mexico City, Mexico) was the contract research organization responsible for conducting the clinical trials. Forty-two healthy Mexican adults (aged 18–55 years) of both sexes were enrolled in the study for a 36 h intern period, admitted to the research site from approximately 16:00 h on day 0 until 08:00 h on day 2. Each study period included a one-day treatment followed by a 14-day washout period. Pharmacokinetic blood sampling and vital sign monitoring were performed at regular intervals. After scheduled activities, subjects were discharged and returned for the 36.00- and 48.00 h post-dosing samples. All adverse events, including duration, severity, causality, and outcomes, were strictly monitored and recorded by the principal investigator and medical staff throughout the study.

### 4.4. Study Population

Participation was voluntary, and written informed consent was obtained from every volunteer before the initiation of any clinical activities. The trial enrolled healthy adults (male and female) aged 18 to 55 with a body mass index (BMI) between 18 and 27 kg/m^2^, as specified in Mexican regulations [24]. Assessment of health status at screening included medical history review, physical examination, 12-lead electrocardiography (ECG), and comprehensive laboratory evaluations—specifically complete blood count (CBC), blood chemistry, urinalysis, toxicology, and pregnancy testing.

At the baseline visit (Day 0), eligibility was reaffirmed through confirmatory pregnancy and alcohol breath testing. Women of reproductive potential were mandated to utilize highly effective contraceptive measures throughout the study timeline. Exclusion criteria comprised pregnancy or lactation, documented history of substance abuse, significant gastrointestinal pathology, or consumption of over-the-counter or prescribed medication within 14 days before drug administration.

### 4.5. Sample Size

Sample size estimation was performed independently for each of the four clinical studies to ensure uniform statistical rigor. The sample size and statistical power were calculated in strict adherence to the NOM-177-SSA1-2013 guideline [24], which mandates that the study be powered based on the pharmacokinetic parameter with the highest anticipated intra-subject coefficient of variation (CV_intra_). Based on an internal pilot study from Axis Clinical Latina, the CV_intra_ for empagliflozin ranged between 6.90% and 18.20% (depending on the evaluated strength), whereas metformin exhibited the highest variability with a maximum CV_intra_ estimated at 24.2% for metformin C_max_ [24]. The calculation incorporated a target statistical power of at least 90%, a significance level of α=0.05, and an expected test-to-reference geometric mean ratio of 0.95 [24], utilizing the two-one-sided tests (TOST) framework via R software version 4.1.2 (Rcmdr package, 2021) appropriate for a standard 2 × 2 crossover design [47,48]. Under these parameters, a minimum of 36 evaluable participants were required per study to robustly establish bioequivalence within the 80.00–125.00% regulatory acceptance range [25]. To conservatively account for potential early withdrawals, dropouts, or protocol deviations of up to 15%, the final enrollment target was increased to 42 subjects per study.

### 4.6. Pharmacokinetic Assessment and Analytical Method

Serial venous blood samples (6 mL) were collected into K_2_EDTA tubes at pre-dose (0 h) and at 22 subsequent time points: 0.25, 0.50, 0.75, 1.00, 1.25, 1.50, 1.75, 2.00, 2.50, 3.00, 3.50, 4.00, 4.50, 5.00, 6.00, 8.00, 10.00, 12.00, 16.00, 24.00, 36.00, and 48.00 h post-dosing. Promptly after collection, the samples were centrifuged at 3500 rpm for 10 min at 4 °C to separate the plasma. The harvested plasma was then transferred into appropriately labeled cryovials and immediately cryopreserved at −70 ± 15 °C until bioanalytical quantification.

Plasma concentrations of empagliflozin and metformin were quantified using high-performance liquid chromatography coupled with tandem mass spectrometry (LC-MS/MS). This method was developed and fully validated by Axis Clinical Latina, S.A. de C.V. (Mexico City, Mexico) in accordance with applicable Mexican regulatory standards and internationally recognized bioanalytical validation guidelines, including those issued by the USA Food and Drug Administration (FDA) [49], and Good Laboratory Practice (GLP).

Analytes and their respective isotopic internal standards (ISs), empagliflozin-d4 and metformin-d6, were extracted from plasma matrices using solid-phase extraction (SPE). Plasma samples were combined with the IS, pretreated, and loaded onto conditioned Strata-X (33 µm) extraction cartridges. Following sequential aqueous and methanolic washes to remove endogenous matrix components, the analytes were eluted from the column with the chromatographic mobile phase before analysis. A 20 µL aliquot of the resulting extract was then injected into the chromatographic system. Chromatographic separation was achieved on a Prominence LC-20AD SIL-20AC HPLC system (SHIMADZU, Kyoto, Japan) equipped with a Kinetex^®^ C_8_ 5 µm, 100 Å, 150 × 4.6 mm column) (Phenomenex, Torrance, CA, USA). The isocratic mobile phase comprised methanol, acetonitrile, and 15 mM ammonium formate in a 70:5:25 (*v*/*v*/*v*) ratio, delivered at a constant flow rate of 0.9 mL/min.

Detection was performed on an API 4000 triple quadrupole mass spectrometer (ABSCIEX, Woodlands, North Region, Singapore) equipped with a TurboIonSpray source operating in positive electrospray ionization (ESI+) mode. The analytes were monitored via multiple reaction monitoring (MRM) using the following specific mass-to-charge (*m*/*z*) transitions: empagliflozin: 468.30 > 355.30, metformin: 130.20 > 71.10, empagliflozin-d4 (IS): 472.20 > 359.20, metformin-d6 (IS): 136.20 > 77.00.

The method demonstrated robust linearity over the calibration ranges of 2.00 to 807.57 ng/mL for empagliflozin and 10.00 to 3017.70 ng/mL for metformin. To continuously monitor assay performance, Quality Control (QC) samples were incorporated into every analytical run. The established QC concentrations were 5.99, 41.12, 132.63, and 309.01 ng/mL for empagliflozin, and 29.90, 311.40, 965.60, and 2299.00 ng/mL for metformin. Comprehensive validation confirmed that the method met all acceptance criteria for selectivity, linearity, precision, accuracy, and robustness. Furthermore, analyte stability was rigorously verified across all operational conditions, including stock solutions, freeze–thaw cycles, autosampler residence, processed samples, and short- and long-term storage configurations. A detailed summary of the bioanalytical method validation, including assessments of linearity, precision, accuracy, selectivity, recovery, matrix effect, sensitivity, and stability, is provided in Supplementary Materials S1, Section S1.2 and Table S5. This validated LC–MS/MS method was used to quantify empagliflozin and metformin plasma concentrations across all four bioequivalence studies evaluated in the present work.

### 4.7. Tolerability and Safety Assessments

Clinical safety and tolerability were rigorously monitored throughout the study. The attending medical staff conducted physical examinations before dosing and immediately before the subjects’ final discharge from the clinical facility.

To ensure continuous participant well-being, vital signs—including blood pressure, heart rate, and body temperature—were recorded at baseline (pre-dose) and at predefined intervals: 2.00, 11.00, 22.00, 36.00, and 48.00 h post-dosing.

The prevalence, severity, and potential causality of adverse events (AEs) associated with the study medications were continuously evaluated. AEs were captured via both spontaneous reporting by the volunteers and direct clinical observation by the principal investigator and medical personnel. All documented events were formally classified by intensity (mild, moderate, or severe), duration, and outcome, ensuring a comprehensive and robust tolerability profile for the evaluated fixed-dose combinations.

### 4.8. Statistical Analysis

Baseline demographic and clinical characteristics of the study cohorts were summarized utilizing standard descriptive statistics. Safety evaluations were conducted on the intention-to-treat (ITT) population, which included all randomized subjects who received at least one dose of the study medications (*n* = 42 per study). Pharmacokinetic (PK) and statistical analyses for bioequivalence were performed exclusively on the per-protocol populations, comprising volunteers who completed both clinical periods and provided evaluable plasma concentration–time profiles without major protocol deviations. Due to participant withdrawals during the clinical phase, the final evaluable per-protocol cohorts consisted of 41, 38, 35, and 37 subjects for Studies A, B, C, and D, respectively.

The primary pharmacokinetic parameters evaluated to establish bioequivalence were the maximum observed plasma concentration (C_max_) and the area under the concentration–time curve from time zero to the last measurable concentration (AUC_0-t_). Furthermore, the total area under the curve extrapolated to infinity (AUC_0-∞_), time to reach maximum concentration (t_max_), elimination rate constant (K_el_), and elimination half-life (t_1/2_) were determined as secondary metrics.

All PK variables were derived via non-compartmental analysis (NCA) using Phoenix^®^ WinNonlin^®^ version 8.4 software (Certara L.P., Princeton, NJ, USA). Peak exposure values (C_max_ and t_max_) were extracted directly from the experimental Cp vs. time profile. The AUC_0-t_ was calculated utilizing the linear trapezoidal rule, while AUC_0-∞_ was extrapolated by adding the Clast/K_el_ quotient to the AUC_0-t_.

To formally evaluate bioequivalence, Schuirmann’s two-one-sided *t*-tests (TOST) approach was applied to the log-transformed primary parameters (C_max_ and AUC_0-t_). An analysis of variance (ANOVA) was additionally executed to evaluate potential sources of variation, including sequence, period, and formulation effects. In compliance with the Mexican regulatory framework (NOM-177-SSA1-2013) [24], 90% confidence intervals (CIs) were constructed for the geometric mean ratios (test/reference) of these parameters. The formulations were deemed bioequivalent if the calculated 90% CIs fell within the accepted regulatory margins of 80.00% to 125.00%.

As an exploratory analysis, metformin dose proportionality was assessed using the power model, ln (PK) = ln(α) + β·ln(dose), fitted by ordinary least-squares linear regression to the log-transformed C_max_, AUC_0-t,_ and AUC_0-∞_ of the reference formulations across the evaluated dose range (500, 850, and 1000 mg) [50,51,52].

Because the four bioequivalence studies were independent, parallel-group studies conducted in different subjects, each subject contributed a single reference observation, and the regression was performed across studies. Both 850 mg studies were included (Study A, co-administered with 5 mg empagliflozin; Study C, with 12.5 mg empagliflozin), and a sensitivity analysis excluding Study C was performed.

Dose proportionality was concluded when the 90% confidence interval (CI) of the slope β was entirely contained within the critical region [1 + ln(θL)/ln(r), 1 +ln(θU)/ln(r)], where r is the ratio of the highest to the lowest dose (1000/500 = 2) and (θL, θU) = (0.80, 1.25); for r = 2 this corresponds to an acceptance interval of [0.678, 1.322] [50]. A slope of β = 1 indicates strict dose proportionality. The power-model dose-proportionality analysis was performed in R version 4.5.3 (R Core Team, R Foundation for Statistical Computing, Vienna, Austria), using the lm and confint functions of the base stats package; figures were generated with the ggplot2 package. Because the studies were not designed to evaluate dose proportionality, this analysis remained exploratory.

## 5. Conclusions

In conclusion, the four immediate-release fixed-dose combination products of empagliflozin/metformin manufactured by Asofarma de México S.A. de C.V. demonstrated bioequivalence to their respective reference products under fed conditions. All primary pharmacokinetic parameters met the regulatory acceptance criteria. Additionally, the consistent pharmacokinetic performance observed for both empagliflozin and metformin across the different dosage strengths further supports the robustness of the fixed-dose combination strategy. An exploratory power-model analysis indicated a modest, less-than-dose-proportional increase in metformin exposure across the 500–1000 mg range (β ≈ 0.77–0.78), consistent with its known saturable absorption; this did not affect the bioequivalence outcomes. The formulations exhibited a favorable safety and tolerability profile; all reported adverse events were non-serious, mild in intensity, and resolved completely. These findings fulfill the bioequivalence requirements established by COFEPRIS and support the therapeutic interchangeability of the evaluated formulations in healthy Mexican subjects.

## Figures and Tables

**Table 1:** Demographic baseline characteristics of study subjects. Continuous variables are presented as mean ± standard deviation (SD), and categorical variables are presented as number of subjects and percentages (n%).

Variable	Study A (5/850 mg), (*n* = 42)	Study B (12.5/500 mg), (*n* = 42)	Study C (12.5/850 mg), (*n* = 42)	Study D (12.5/1000 mg), (*n* = 42)
**Gender, n (%)**				
Male	21 (50.0%)	23 (54.8%)	25 (59.5%)	16 (38.1%)
Female	21 (50.0%)	19 (45.2%)	17 (40.5%)	26 (61.9%)
**Age (years)**	36.30 ± 10.20	35.00 ± 11.00	36.40 ± 12.40	37.90 ± 12.30
**Weight (kg)**	66.40 ± 9.70	65.00 ± 10.90	63.50 ± 10.60	63.30 ± 10.00
**Height** **(m)**	1.65 ± 0.09	1.60 ± 0.10	1.65 ± 0.09	1.62 ± 0.09
**BMI (kg/m^2^)**	24.30 ± 2.00	24.00 ± 2.30	23.30 ± 2.72	24.00 ± 2.10

SD: standard deviation; n: number of subjects; BMI: body mass index; kg: kilograms; m: meters; mg: milligrams.

**Table 2:** Primary and secondary pharmacokinetic parameters of empagliflozin and metformin following oral administration of the test and reference fixed-dose combinations under fed conditions. Data are presented as mean ± standard deviation (SD).

PK Parameter	Empagliflozin (Test)	Empagliflozin (Reference)	Metformin (Test)	Metformin (Reference)
Study A (5/850 mg)				
C_max_ (ng/mL)	69.87 ± 21.15	72.83 ± 23.25	1662.40 ± 398.60	1620.78 ± 405.92
t_max_ (h)	4.27 ± 1.91	4.11 ± 1.85	3.47 ± 1.51	3.57 ± 1.41
AUC_0-t_ (ng·h/mL)	602.45 ± 123.52	616.35 ± 137.79	12,747.12 ± 2610.88	12,534.53 ± 2540.88
AUC_0-∞_ (ng·h/mL)	639.27 ± 130.20	650.20 ± 138.09	12,961.73 ± 2627.80	12,719.16 ± 2579.66
t_1/2_ (h)	6.73 ± 2.77	6.44 ± 1.46	7.27 ± 6.70	7.10 ± 5.20
Study B (12.5/500 mg)				
C_max_ (ng/mL)	175.67 ± 42.86	181.40 ± 43.16	1003.83 ± 198.13	1035.38 ± 197.61
t_max_ (h)	4.28 ± 1.43	4.59 ± 1.43	3.95 ± 1.70	3.95 ± 1.53
AUC_0-t_ (ng·h/mL)	1624.79 ± 306.56	1693.04 ± 292.83	8204.84 ± 1571.07	8244.03 ± 1412.65
AUC_0-∞_ (ng·h/mL)	1679.56 ± 316.72	1742.32 ± 301.04	8333.56 ± 1589.13	8440.30 ± 1571.63
t_1/2_ (h)	8.61 ± 2.56	8.89 ± 2.00	5.19 ± 2.13	6.07 ± 7.87
Study C (12.5/850 mg)				
C_max_ (ng/mL)	197.19 ± 52.16	194.62 ± 48.40	1662.70 ± 395.44	1681.17 ± 401.40
t_max_ (h)	3.78 ± 1.40	4.08 ± 1.87	3.44 ± 1.52	3.68 ± 1.41
AUC_0-t_ (ng·h/mL)	1733.41 ± 339.58	1756.70 ± 298.42	13,068.08 ± 3843.43	13,289.47 ± 4041.19
AUC_0-∞_ (ng·h/mL)	1771.54 ± 342.54	1798.17 ± 297.90	13,248.26 ± 3806.50	13,490.78 ± 4035.56
t_1/2_ (h)	7.33 ± 1.74	7.81 ± 1.91	6.22 ± 3.29	6.52 ± 5.71
Study D (12.5/1000 mg)				
C_max_ (ng/mL)	194.78 ± 48.68	192.22 ± 46.77	1768.30 ± 428.12	1713.05 ± 385.76
t_max_ (h)	4.10 ± 1.32	4.16 ± 1.78	4.04 ± 1.30	3.76 ± 1.71
AUC_0-t_ (ng·h/mL)	1651.53 ± 351.96	1614.99 ± 334.33	14,834.58 ± 3817.22	14,212.20 ± 3691.61
AUC_0-∞_ (ng·h/mL)	1688.27 ± 351.97	1649.86 ± 337.38	15,023.66 ± 3815.50	14,413.57 ± 3718.26
t_1/2_ (h)	7.68 ± 2.10	7.52 ± 2.05	6.62 ± 3.27	6.67 ± 3.78

C_max_: maximum observed plasma concentration; t_max_: time to reach maximum plasma concentration; AUC_0-t_: area under the plasma concentration–time curve from time zero to the last quantifiable concentration; AUC_0-∞_: area under the plasma concentration–time curve from time zero extrapolated to infinity; t_1/2_: terminal elimination half-life.

**Table 3:** Geometric mean ratios and 90% confidence intervals (CIs) of the primary pharmacokinetic parameters for empagliflozin and metformin.

Study/Analyte	Parameter	Geometric Mean Ratio (%)	90% CI (LL–UL)	CV_intra_ (%)	TOST p1 (<0.80)	TOST p2 (>1.25)	Power
Study A (5/850 mg)							
Empagliflozin	C_max_	96.45	90.18–103.16	18.20	<0.001	<0.001	1.00
	AUC_0-t_	98.02	94.37–101.81	10.20	<0.001	<0.001	1.00
Metformin	C_max_	102.96	98.43–107.69	12.10	<0.001	<0.001	1.00
	AUC_0-t_	101.79	97.18–106.62	12.50	<0.001	<0.001	1.00
Study B (12.5/500 mg)							
Empagliflozin	C_max_	97.20	91.94–102.77	14.40	<0.001	<0.001	1.00
	AUC_0-t_	95.91	93.38–98.51	6.90	<0.001	<0.001	1.00
Metformin	C_max_	97.05	92.58–101.73	12.20	<0.001	<0.001	1.00
	AUC_0-t_	99.27	94.85–103.91	11.80	<0.001	<0.001	1.00
Study C (12.5/850 mg)							
Empagliflozin	C_max_	101.42	95.47–107.74	15.00	<0.001	<0.001	1.00
	AUC_0-t_	98.25	95.34–101.25	7.50	<0.001	<0.001	1.00
Metformin	C_max_	98.78	94.19–103.59	11.80	<0.001	<0.001	1.00
	AUC_0-t_	98.53	94.06–103.21	11.50	<0.001	<0.001	1.00
Study D (12.5/1000 mg)							
Empagliflozin	C_max_	100.62	94.53–107.10	15.90	<0.001	<0.001	1.00
	AUC_0-t_	101.83	98.13–105.66	9.40	<0.001	<0.001	1.00
Metformin	C_max_	103.11	98.89–107.51	10.60	<0.001	<0.001	1.00
	AUC_0-t_	104.45	99.25–109.92	13.00	<0.001	<0.001	1.00

LL: lower limit; UL: upper limit. Schuirmann’s two one-sided tests (TOST). C_max_: maximum observed plasma concentration; AUC_0-t_: area under the plasma concentration–time curve from time zero to the last quantifiable concentration.

**Table 4:** Mean metformin pharmacokinetic exposure and dose-normalized exposure across the evaluated dose range in the reference formulations.

Study	Metformin Dose (mg)	Empagliflozin Dose (mg)	n	C_max_ (ng/mL)	C_max_/Dose	AUC_0-t_ (ng⋅h/mL)	AUC_0-t_/Dose
B	500	12.5	38	1035.4	2.071	8243.3	16.48
A	850	5.0	41	1620.8	1.907	12,530.4	14.74
C	850	12.5	35	1681.2	1.978	13,288.1	15.63
D	1000	12.5	37	1713.0	1.713	14,210.3	14.210

n: number of subjects; C_max_: maximum observed plasma concentration; AUC_0-t_: area under the plasma concentration–time curve from time zero to the last quantifiable concentration. Dose-normalized parameters (C_max_/dose and AUC_0-t_/dose) were calculated by dividing the mean pharmacokinetic exposure by the respective administered metformin dose.

**Table 5:** Power-model assessment of metformin dose proportionality (reference formulations).

Analysis	Parameter	n	β (Slope)	90% CI	Acceptance Region (r = 2)	Conclusion
All reference data	C_max_	151	0.771	0.656–0.887	0.678–1.322	Not within
All reference data	AUC_0-t_	151	0.780	0.660–0.900	0.678–1.322	Not within
All reference data	AUC_0-∞_	151	0.768	0.648–0.888	0.678–1.322	Not within
Excluding Study C	C_max_	116	0.748	0.631–0.865	0.678–1.322	Not within
Excluding Study C	AUC_0-t_	116	0.766	0.652–0.879	0.678–1.322	Not within
Excluding Study C	AUC_0-∞_	116	0.754	0.639–0.868	0.678–1.322	Not within

n: number of observations; C_max_: maximum observed plasma concentration; AUC_0-t_: area under the plasma concentration–time curve from time zero to the last quantifiable concentration; AUC_0-∞_: area under the plasma concentration–time curve from time zero extrapolated to infinity; CI: confidence interval; b: power-model slope estimate; r: ratio of the highest to the lowest evaluated dose (1000 mg/500 mg).

**Table 6:** Summary of adverse events (AEs) by study and treatment.

Study/Dosage	Adverse Event (AE)	Test (n)	Reference (n)	Total Events
Study A 5/850 mg	Nausea	3	7	10
	Headache	3	3	6
	Dyspepsia	1	1	2
	Vomiting	1	1	2
	Loose stools	1	0	1
	Subtotal Study A	9	12	21
Study B 12.5/500 mg	Headache	2	5	7
	Nausea	1	2	3
	Dyspepsia	1	1	2
	Loose stools	1	2	3
	Subtotal Study B	5	10	15
Study C 12.5/850 mg	Headache	5	4	9
	Nausea	2	1	3
	Upper abdominal pain	1	1	2
	Dyspepsia	1	0	1
	Vomiting	1	0	1
	Dizziness	0	1	1
	Loose stools	1	3	4
	Pyrexia	0	1	1
	Subtotal Study C	11	11	22
Study D 12.5/1000 mg	Headache	8	7	15
	Nausea	10	4	14
	Dyspepsia	1	0	1
	Dysmenorrhea	1	0	1
	Dizziness	1	0	1
	Skin rash	1	0	1
	Vomiting	1	0	1
	Medical device site rash	1	0	1
	Loose stools	0	1	1
	Subtotal Study D	24	12	36
Total Program	All adverse events	49	45	94

Values represent the absolute frequency of reported adverse events mapped per treatment arm (test vs. reference) across all four clinical trials (Studies A–D). All events were classified as mild or moderate in intensity and resolved completely.

## Data Availability

The data presented in this study are available on request from the corresponding author due to confidentiality agreements with the sponsor.

## Supplementary Materials

The following supporting information can be downloaded at: https://www.mdpi.com/article/10.3390/ph19081200/s1, Table S1: Measured concentrations and variability across three independent calibration runs for the evaluation of method linearity of Empagliflozin in human plasma; Table S2: Calibration curve parameters for Empagliflozin; Table S3: Measured concentrations and variability across three independent calibration runs for the evaluation of method linearity of Metformin in human plasma; Table S4: Calibration curve parameters for Metformin; Table S5: Summary of bioanalytical method validation results for Empagliflozin and Metformin; Figure S1: Representative calibration curve obtained using analyte-to-internal standard peak area ratios, showing the linear relationship between concentration and response for Empagliflozin in human plasma; Figure S2: Representative calibration curve obtained using analyte-to-internal standard peak area ratios, showing the linear relationship between concentration and response for Metformin in human plasma.

## Abbreviations

The following abbreviations are used in this manuscript:

ADA	American Diabetes Association
AEs	Adverse events
ANOVA	Analysis of variance
API	Active pharmaceutical ingredient
ASCVD	Atherosclerotic cardiovascular disease
AUC	Area under the curve
AUC_0-t_	Area under the plasma concentration–time curve from time zero to the last quantifiable concentration
AUC_0-∞_	Area under the plasma concentration–time curve from time zero extrapolated to infinity
BCRP	Breast cancer resistance protein
BCS	Biopharmaceutical Classification System
BMI	Body mass index
CBC	Complete blood count
CI	Confidence interval
C_max_	Peak plasma concentration
CoA	Certificate of Analysis
COFEPRIS	Federal Commission for the Protection against Sanitary Risk
CONSORT	Consolidated Standards of Reporting Trials
Cp	Plasma concentration
CRO	Contract Research Organization
CVintra	Intra-subject coefficient of variation
CYP	Cytochrome P450
DM	Diabetes mellitus
ECG	Electrocardiogram
ESI^+^	Positive electrospray ionization
f_2_	Similarity factor
FDA	Food and Drug Administration
FDC	Fixed-dose combination
FEUM	Farmacopea de los Estados Unidos Mexicanos
GCP	Good Clinical Practice
GLP	Good Laboratory Practice
HbA1c	Glycated hemoglobin
HPLC	High-performance liquid chromatography
ICH	International Council for Harmonization
IR	Immediate release
IS	Internal standard
ITT	Intention-to-treat
IVIVC	In vitro–in vivo correlation
K_el_	Terminal elimination rate constant
LC-MS/MS	Liquid chromatography coupled with tandem mass spectrometry
LL	Lower limit
*m*/*z*	Mass-to-charge ratio
MRM	Multiple reaction monitoring
NCA	Non-compartmental analysis
OCT	Organic cation transporter
OTC	Over-the-counter
P-gp	P-glycoprotein
PES	Polyethersulfone
PK	Pharmacokinetics
PMAT	Plasma membrane monoamine transporter
PP	Per-protocol
QC	Quality control
SAEs	Serious adverse events
SD	Standard deviation
SGLT2	Sodium–glucose cotransporter-2
SPE	Solid-phase extraction
T2D	Type 2 diabetes
T2DM	Type 2 diabetes mellitus
t_1/2_	Elimination half-life
t_max_	Time to reach peak plasma concentration
TOST	Two one-sided *t*-tests
UGT	Uridine diphosphate glucuronosyltransferase
UL	Upper limit
USP	United States Pharmacopeia
UV	Ultraviolet
Vd	Apparent volume of distribution
WMA	World Medical Association
